# Dermatitis during adjuvant irradiation for breast cancer (DAI-BREAC): a randomized controlled trial investigating whether grade ≥2 dermatitis during radiotherapy for breast cancer can be reduced by a mobile application that reminds patients to perform skin care

**DOI:** 10.1186/s13063-025-08800-2

**Published:** 2025-03-20

**Authors:** Dirk Rades, Carlos Andres Narvaez-Wolf, Liesa Dziggel, Christian Staackmann, Maike Radtke, Carmen Timke, Charlotte Kristiansen, Marciana-Nona Duma, Nathan Y. Yu, Stefan Janssen

**Affiliations:** 1 Department of Radiation Oncology, University of Lübeck, Lübeck, Germany; 2 Department of Radiation Oncology, University Medical Center Schleswig-Holstein, Campus Lübeck, Lübeck, Germany; 3 Department of Radiotherapy, Malteser Hospital St. Franziskus Flensburg, Flensburg, Germany; 4 Department of Oncology, Vejle Hospital, University Hospital of Southern Denmark, Vejle, Denmark; 5 Department of Radiotherapy, Helios Hospital Schwerin, Schwerin, Germany; 6 Department of Radiation Oncology, Mayo Clinic, Phoenix, AZ USA

**Keywords:** Breast cancer, Adjuvant radiotherapy, Grade ≥2 radiation dermatitis, Mobile application

## Abstract

**Background:**

Radiotherapy of breast cancer can be associated with dermatitis. Grade ≥2 radiation dermatitis can be painful and impair the patients’ quality of life. To reduce the risk of this complication, patients have to perform skin care several times each day. This may require a considerable level of compliance. This randomized DAI-BREAC trial investigates whether a mobile application reminding the patients four times per day to perform skin care (reminder app) will contribute to reduction of grade ≥2 radiation dermatitis.

**Methods:**

This multinational, randomized, active-controlled, parallel-group, multicenter trial compares standard skin care supported by a reminder app (Arm A) versus standard skin care alone (Arm B) regarding grade ≥2 radiation dermatitis in patients receiving adjuvant radiotherapy for breast cancer. The effect of the app will be considered clinically relevant, if the rate of grade ≥2 radiation dermatitis is reduced from 25.4% (rate identified in a preceding study) to 15%. A total of 134 patients per arm including drop-outs are required. Secondary aims include pain (visual analogue scale), patient satisfaction with the app (questionnaire), impact of the app on the use of health technology (questionnaire), and benefit from support (coaching) by staff members regarding the use of the app (questionnaire).

**Discussion:**

If the reminder app contributes to a decrease of grade ≥2 radiation dermatitis in patients irradiated for breast cancer, it will likely become a useful instrument for these patients.

**Trial registration:**

Clinicaltrials.gov (NCT06483477; URL: https://clinicaltrials.gov/show/NCT06483477). Registered on 1st of July, 2024. First patient was included in December 2024.

## Administrative information

Note: the numbers in curly brackets in this protocol refer to SPIRIT checklist item numbers. The order of the items has been modified to group similar items (see http://www.equator-network.org/reporting-guidelines/spirit-2013-statement-defining-standard-protocol-items-for-clinical-trials/).

**Table:** 

Title {1}	**Dermatitis during adjuvant irradiation for breast cancer (DAI-BREAC): Grade ≥2 radiation dermatitis in breast cancer patients with or without a mobile application (reminder app)**
Trial registration {2a and 2b}	NCT06483477, clinicaltrials.gov
Protocol version {3}	21-JUN-2024, version 1.2
Funding {4}	As part of the project HeAT, the DAI-BREAC trial was funded by the European Regional Development Fund through the Interreg Deutschland-Danmark program, reference: 01-1-23 2.
Author details {5a}	(1) Dirk Rades, Department of Radiation Oncology, University of Lübeck, Lübeck, Germany; dirk.rades@uksh.de (2) Carlos Andres Narvaez-Wolf, Department of Radiation Oncology, University Medical Center Schleswig-Holstein, Campus Lübeck, Lübeck, Germany; Carlos.Narvaez-Wolf@uksh.de (3) Liesa Dziggel, Department of Radiation Oncology, University Medical Center Schleswig-Holstein, Campus Lübeck, Lübeck, Germany; liesa.dziggel@uksh.de (4) Christian Staackmann, Department of Radiation Oncology, University Medical Center Schleswig-Holstein, Campus Lübeck, Lübeck, Germany; christian.staackmann@uksh.de (5) Maike Radtke, Department of Radiation Oncology, University Medical Center Schleswig-Holstein, Campus Lübeck, Lübeck, Germany; maike.radtke@uksh.de (6) Carmen Timke, Department of Radiotherapy, Malteser Hospital St. Franziskus Flensburg, Flensburg, Germany; carmen.timke@malteser.org (7) Charlotte Kristiansen, Department of Oncology, Vejle Hospital, University Hospital of Southern Denmark, Vejle, Denmark; charlotte.kristiansen@rsyd.dk (8) Marciana-Nona Duma, Department of Radiotherapy, Helios Hospital Schwerin, Schwerin, Germany; Marciana.Duma@helios-gesundheit.de (9) Nathan Y. Yu, Department of Radiation Oncology, Mayo Clinic, Phoenix, AZ, USA; Yu.Nathan@mayo.edu (10) Stefan Janssen, Department of Radiation Oncology, University of Lübeck, Lübeck, Germany; stefan.janssen@uksh.de
Name and contact information for the trial sponsor {5b}	Sponsor: University Medical Center Schleswig-Holstein (UKSH), Campus Lübeck, Ratzeburger Allee 160, 23538 Lübeck, Germany Coordinating Investigator (contact) Prof. Dr. Dirk Rades Department of Radiation Oncology, University of Lübeck Ratzeburger Allee 160, 23562 Lübeck, Germany. Tel.: +49-(0)451-500-45400 Fax: +49-(0)451-500-45404 Email: dirk.rades@uksh.de
Role of sponsor {5c}	The sponsor and the funding body have no role in the design of the study, in collection, analysis and interpretation of the data and in the writing of the manuscript.

## Introduction

### Background and rationale {6a}

Many breast cancer patients receive adjuvant radiotherapy following breast conserving surgery or mastectomy [1, 2]. Radiotherapy of breast cancer can be associated with acute toxicities including radiation dermatitis. Grade ≥2 dermatitis can be painful and impair the patients’ quality of life [3, 4]. To reduce the risk of radiation dermatitis, the patients are asked to perform skin care several times per day during their radiotherapy course and afterwards, which may require a high level of discipline. In a randomized trial of patients irradiated for head-and-neck cancer, daily reminders by medical staff members to perform skin care appeared to increase the patients’ compliance resulting in less acute dermatitis [5]. Subsequently, the question has been raised whether the daily reminders by medical staff members could be replaced by a mobile application (reminder app) reminding the patients several times daily to perform skin care. This hypothesis was investigated in another prospective trial that compared standard skin care supported by a reminder app and standard skin care alone in patients with head-and-neck cancer [6]. That trial was prematurely terminated because only 56 of 168 patients were recruited due to slow accrual. At this stage, the reminder app has led to a non-significant reduction of grade ≥2 dermatitis. Reasons for slow accrual included the facts that many patients screened for eligibility had no smartphone or refused to participate in the trial.

These reasons were considered to be more pronounced in patients with head-and-neck cancer than in patients with breast cancer. In a previous prospective study, sleep disorders during a radiotherapy course and the role of smartphones or tablets in breast cancer patients were investigated [7]. The proportion of screened patients participating in this trial was significantly greater than 90%. Therefore, it was decided to additionally investigate a reminder app in a cohort of breast cancer patients. In the present DAI-BREAC trial, a reminder app will be prospectively tested that reminds breast cancer patients four times each day to perform the required skin care. This will likely contribute to the reduction of grade ≥2 radiation dermatitis in these patients.

### Objectives {7}

The main goal of this randomized trial is to evaluate whether standard skin care supported by a reminder app is superior to standard skin care alone with respect to prevention of grade ≥2 radiation dermatitis in patients receiving adjuvant radiotherapy for breast cancer. Secondary aims include pain (radiation fields), patient satisfaction with the reminder app, impact of the reminder app on the use of health technology, and benefit from support (coaching) by staff members regarding the use of the reminder app.

### Trial design {8}

This is a multinational, randomized, active-controlled, open-label, multicenter, parallel-group trial, which compares the following treatments of radiation related skin toxicity in patients with breast cancer:

Standard skin care supported by a reminder app (Arm A) vs. standard skin care alone (Arm B).

## Methods: Participants, interventions and outcomes

### Study setting {9}

The study will be performed in Germany (one university hospital, two academic teaching hospitals, one private practice) and Denmark (one university hospital). Participating sites can be seen at clinicaltrials.gov.

### Eligibility criteria {10}

Inclusion criteria

- 1.Histologically proven invasive breast cancer
- 2.Indication for adjuvant hypo-fractionated radiotherapy [8, 9]
- 3.Possession of and ability to use a smartphone
- 4.Female gender
- 5. Age ≥18 years
- 6.Written informed consent
- 7.Capacity of the patient to consent

Exclusion criteria

- 1.Pregnancy, Lactation
- 2.Expected non-compliance

Male patients were not included, since male breast cancer is increasingly considered a separate tumor entity with a biological behavior different from female breast cancer [10]. Radiation dermatitis will be assessed by an experienced observer (specially trained nurse, technician, or physician) different from the person who performs the routine visit of the patient (“blinded observer concept”), at the start of radiotherapy, weekly during the course of radiotherapy, and at the end of radiotherapy.

### Who will take informed consent? {26a}

Informed consent will be taken by specially trained physicians registered as investigators for this trial.

### Additional consent provisions for collection and use of participant data and biological specimens {26b}

Additional collection and use of participant data or biological specimens are not planned.

### Interventions

#### Explanation for the choice of comparators {6b}

In a previous randomized trial of patients irradiated for head-and-neck cancer, daily reminders by staff members regarding skin care likely improved the patients’ compliance, which resulted in less radiation dermatitis [5]. A subsequent randomized trial investigating whether the daily reminders could be replaced by a mobile application (reminder app) failed to showed a significant impact, possibly because the trial was prematurely terminated due to slow accrual [6]. Since breast cancer patients often show a great interest to participate in a clinical trial, it was felt that the recruitment for a randomized trial investigating the impact of a reminder app on grade ≥2 radiation dermatitis would be more successful in this patient group [7].

### Intervention description {11a}

#### Standard skin care

In both the experimental arm (A) and the control arm (B) standard skin care has to be performed by the patient from the start of radiotherapy. The patients are supposed to perform the skin care four times a day. Standard skin care may vary at the participating centers. At the University of Lübeck, it includes fatty cream with 2-10% urea (fatty cream alone, if patients do not tolerate urea) and, in case of pruritus, addition of mometasone furoate cream.

#### Reminder App

In addition, patients of Arm A are supported by a Reminder App, which is developed by the professional company Nextlabel OHG from Lübeck. The purpose of the app is to remind the patients in an intuitive, unobtrusive and supportive way to perform skin care. By default, patients are reminded four times a day, but they will also be able to define a notification schedule that best suits their personal needs. The patients may postpone each required care procedure for up to 2 hours. With each procedure, the patients are guided through the skin care with simple and self-explanatory illustrations. To increase the patients’ motivation, they will additionally earn points for regular and punctual performed care procedures. The patients receive a hand-out including instructions how to properly perform skin care, but they can also find an information page within the application that includes similar instructions.

### Criteria for discontinuing or modifying allocated interventions {11b}

In case of grade ≥2 moist desquamation or grade ≥3 radiation dermatitis, each day antiseptic agents will be administered for wound cleansing followed by administration of silicon or calcium alginate bandage in patients of both arms. This treatment will be continued until moist desquamation radiation disappears and radiation dermatitis improves to grade 2.

### Strategies to improve adherence to interventions {11c}

Patients will be seen by staff members (radiotherapy technologists) of the treating Department of Radiation Oncology during each treatment session and additionally by investigators of the trial once a week.

### Relevant concomitant care permitted or prohibited during the trial {11d}

Please, refer to 11 b. If required, any type of concomitant care and interventions are permitted during the trial for treatment of other radiotherapy- or radio-chemotherapy-related toxicities and co-morbidities not related to radiotherapy or radio-chemotherapy.

### Provisions for post-trial care {30}

Following the end of radiotherapy (end of study), the study participants receive the standard follow-up program for breast cancer patients. Harm from trial participation is not expected, since all participating patients receive the same anticancer treatment as they would have received if not participating.

### Outcomes {12}

Primary endpoint is the rate of grade ≥2 radiation dermatitis (CTCAE v5.0) at the end of radiotherapy [11].

In addition, the following endpoints will be evaluated:

- 1.Pain (radiation fields): Evaluation prior to, weekly during, and at the end of radiotherapy
- 2.Patient satisfaction with the reminder app (Arm A): Evaluation at the end of radiotherapy
- 3.Impact of the reminder app on the use of health technology (Arm A): Evaluation at the end of radiotherapy
- 4.Benefit from support (coaching) by staff members (Arm A): Evaluation at the end of radiotherapy

### Participant timeline {13}

**Fig 1:**
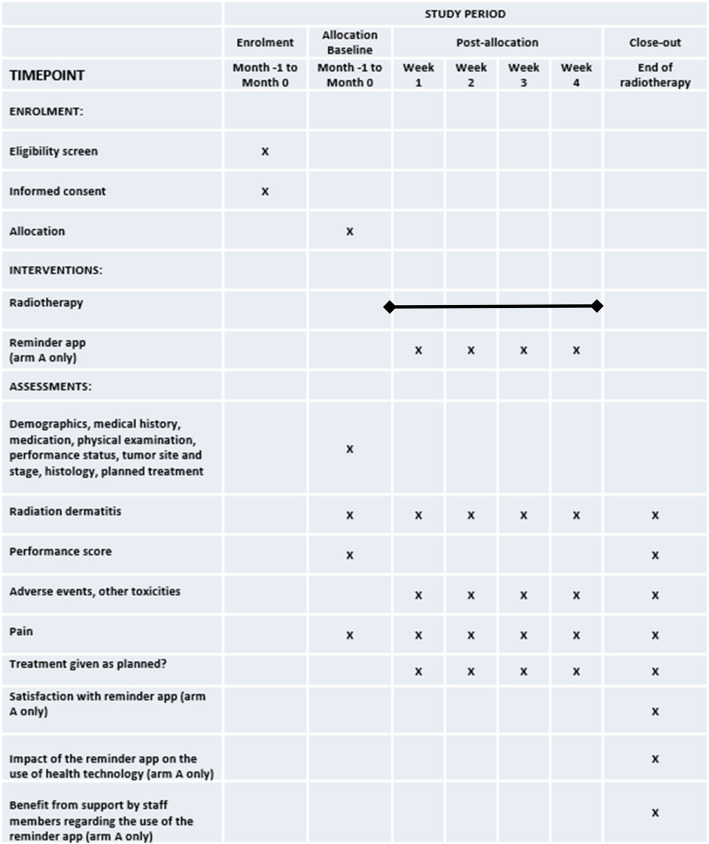
Schedule of enrolment, interventions and assessments

The five centers that are planned to participate aim to include an average of 27 patients per year. Thus, the recruitment of all 268 patients should be completed within 24 months. Since the treatment period will be 3 to 4 weeks, the total running time for the trial is expected to be 25 months. Regarding the timeline of trial procedures and assessments, please see Figure 1.

### Sample size {14}

The null hypothesis of equal rates of grade ≥2 skin toxicity is tested against the two-sided alternative hypothesis of different rates. Based on this hypothesis system, the sample size required for this trial is calculated considering the following assumptions:

- ● A Chi-square Test will be applied
- ● The two-sided significance level is set to 5%
- ● In patients treated with hypo-fractionated radiotherapy for breast cancer, a retrospective pre-study suggested a rate of grade ≥2 dermatitis of 25.4% if standard skin care alone was administered [12].
- ● Based on these retrospective data, a rate of grade ≥2 skin toxicity of approximately 30% was assumed in the reference group of a prospective trial, i.e. in patients receiving standard skin care alone for radiation dermatitis.
- ● The impact of the Reminder App will be considered to be clinically relevant, if the rate of grade ≥2 dermatitis can be reduced to 15%.
- ● The power to yield statistical significance if the difference in rates is in fact 15% is set to 80%.

Based on these assumptions, 131 patients are required per study arm within the Full Analysis Set. Considering that 2% of patients will not qualify for this set, a total of 268 patients should be randomized.

### Recruitment {15}

A total of five contributing centers are planned to participate, who aim to include an average of 27 patients per year. The recruitment of all 268 patients should be completed within 24 months.

### Assignment of interventions: allocation

#### Sequence generation {16a}

The patient identification number will consist of four digits (one digit for the contributing center and three digits for the consecutive patient number). After registration, patients will be randomized in a 1:1 ratio to receive either standard skin care supported by a reminder app (Arm A) or standard skin care (Arm B) for treatment of radiation related skin toxicity. Allocation of a patient to a treatment arm will be performed in the form of a stratified block-randomization with random block size using an electronic case report form (eCRF).

Stratification will be done using the following prognostic factors:

- 1.Treatment volume: Breast or chest wall alone vs. breast or chest wall plus lymph nodes
- 2.Radiation boost: Yes vs. no
- 3.At least one risk factor of dermatitis: Yes vs. no (Risk factors include chronic inflammatory disease, significant cardiovascular disease, and smoking history of >10 pack years.)

The corresponding document will be kept at the institution which performs the randomization until the end of the study. Afterwards, the original randomization list will be kept in the trial master file at the trial center of the coordinating investigator for a minimum of 10 years after the final report. The randomization will be performed via eCRF centrally by an external company using its standard software. The randomization process is based on this company’s standard operating procedures (SOPs). Once the randomization is allocated to the patient it cannot be changed.

### Concealment mechanism {16b}

Allocation of a patient to a treatment arm will be performed using an eCRF.

### Implementation {16c}

Generation of the allocation sequence, enrollment of participants and assignment of participants to interventions will be performed by specially trained physicians registered as investigators for this trial.

## Assignment of interventions: Blinding

### Who will be blinded {17a}

Data analysts and statisticians will be blinded. Moreover, the person assessing radiation dermatitis will be different from the person who performs the routine visit of the patient (“blinded observer concept”),

### Procedure for unblinding if needed {17b}

Each investigator trained for the trial and listed in the delegation log has an individual password protected access to the eCRF, where the information regarding the patient’s allocation is available.

### Data collection and management

#### Plans for assessment and collection of outcomes {18a}

The following parameters will be recorded at the start of the trial: medical history including concomitant diseases (particularly chronic inflammatory disease, cardiovascular disease, and smoking habits), medication including systemic anticancer treatment, physical examination, age, date of birth, gender, body height and body weight, performance status (ECOG-PS [13]), tumor side (affected breast) and location, stage, histology, hormone receptor status, HER2 status, Ki-67 labeling index, triple-negativity, planned total radiation dose, dose per fraction, number of fractions, radiation boost, treatment volume (breast alone, chest wall alone, breast plus lymph nodes, chest wall plus lymph nodes), radiation technique, deep-inspiration breath hold (DIBH) technique, and pain score including the intake of analgesics.

The following parameters will be assessed during the course of the trial:

- 1. Radiation dermatitis: Assessed by an observer different from the person who performs the routine visit of the patient at the start of, weekly during, and at the end of radiotherapy (CTCAE v5.0) [11] .
- 2. Pain (skin within radiation fields): Assessed prior to, weekly during, and at the end of radiotherapy (visual analogue scale). Furthermore, intake of analgesics will be documented.
- 3. Adverse events other than radiation dermatitis will be assessed on an ongoing basis (CTCAE v5.0) [11].
- 4. Eastern Cooperative Oncology Group (ECOG): Evaluated prior to and at the end of radiotherapy [13].
- 5. Physical examination: Performed prior to, weekly during radiotherapy, and at the end of radiotherapy.
- 6. Patient satisfaction with the reminder app, its impact on the use of health technology, and benefit from support (coaching) by staff members: Assessed with specific questionnaires at the end of radiotherapy.

The timeline of trial procedures and assessments is given in Figure 1.

### Plans to promote participant retention and complete follow-up {18b}

The last day of radiotherapy is also the end of the follow up period. Patients are seen at least five days per week by medical staff members. Thus, it is unlikely that patients will be lost to follow up.

### Data management {19}

All data relating to patients will be recorded in a pseudonymous way. Each patient will be identifiable only by the unique patient number, year of birth and gender. A patient identification list will be kept only in the relevant trial centers and not forwarded to the sponsor. Data collection will be done using eCRFs. The data will be handled according to the General Data Protection Regulation (GDPR). The data will be included in one data base and analyzed in accordance with a pre-defined statistical analysis plan (SAP). Originals of key trial documents including documentation sheets will be kept at the trial headquarters (sponsor responsible for the trial) for at least 10 years after the final report. The principal investigator/head of the trial center will keep all administrative documents, the patient identification list, signed informed consent forms, copies of the documentation sheets, and general trial documentation (protocol, amendments) for the abovementioned period. Original patient data must also be kept for the length of time stipulated for the trial centers, but not for less than 10 years.

### Confidentiality {27}

Data will be collected in accordance with the regulations set out in the Data Protection Act. All findings from the clinical trial will be stored on electronic data storage devices and treated with utmost confidentiality. Organization measures have been taken in order to prevent the data from being communicated to unauthorized persons. Patients will only be identified via their individual patient numbers throughout the entire documentation and evaluation phase and their full name will not be used.

### Plans for collection, laboratory evaluation and storage of biological specimens for genetic or molecular analysis in this trial/future use {33}

Laboratory evaluation and storage of biological specimens for genetic or molecular analysis in this trial or for future use are not planned.

### Statistical methods

#### Statistical methods for primary and secondary outcomes {20a}

All data recorded in the eCRFs describing the study population and toxicity will be analyzed descriptively. Categorical data will be presented in contingency tables with frequencies, percentages and their 95% confidence intervals. Continuous data will be summarized with at least the following: frequency (n), median, quartiles, mean, standard deviation (standard error), minimum and maximum. Number of patients with protocol deviations and listings describing the deviations will be provided. In general, chi-square tests will be used to compare percentages in a two-by-two contingency table, replaced by Fisher´s exact test if the expected frequency in at least one cell of the associated table is less than 5. Stratified two-by-two contingency tables will be analyzed using Cochran-Mantel-Haenszel tests. Logistic regression models serve as multivariable methods for binary endpoint data. Comparison of ordinal variables between treatment arms will be performed using the asymptotic Wilcoxon-Mann-Whitney test, replaced by its exact version in case of ordinal categories with small number of categories and/or sparse data within categories. Any shift in location of quantitative variables between study groups will be performed with Wilcoxon-Mann-Whitney tests. Time-to-event data will be analyzed by Kaplan-Meier methods, when merely non-informative censoring occurs. For statistical comparison, the log rank-test will be provided supplemented by multivariate Cox proportional hazards models. The data analysis will be performed according to the SAP finalized prior to database lock and prior to any statistical analysis.

### Interim analyses {21b}

An interim analysis is not planned at this stage.

### Methods for additional analyses (e.g. subgroup analyses) {20b}

Elderly patients aged ≥65 years will be compared to younger patients aged <65 years to identify potential differences between both age groups and the need for support regarding the use of the reminder app. Standard statistical tests serve as a tool for exploratory comparison of age groups.

### Methods in analysis to handle protocol non-adherence and any statistical methods to handle missing data {20c}

The Full Analysis Set includes all randomized patients who have started either therapy with arm A or with arm B and provide any data on the primary endpoint. The Full Analysis Set will be analyzed according to the Intention-to-Treat principle, i.e., patients will be analyzed in their initial group of randomization.

The Per Protocol Set includes all patients of the Full Analysis Set excluding patients in case of administration of less than 75% of the planned radiation dose if the reason for discontinuation was any other than death or unacceptable toxicity and/or in case of >50% missing data regarding the primary study endpoint. All patients in the Per Protocol Set will be analyzed within their group of actual treatment received.

### Plans to give access to the full protocol, participant level-data and statistical code {31c}

Many details of the study protocol including statistical considerations are available at clinicaltrials.gov. The full protocol and individual participant data will not be shared.

### Oversight and monitoring

#### Composition of the coordinating center and trial steering committee {5d}

The staff of the coordinating center includes the principle investigator, his deputy, and more than ten sub-investigators plus a study nurse/secretary. There is no specific steering committee for this trial. The trial is part of a subgoal of the Interreg-project HeAT including five trials. Senior staff members of the project and network partners involved in the subgoal and the corresponding trials meet twice a year.

#### Composition of the data monitoring committee, its role and reporting structure {21a}

A data monitoring committee is not required, since all patients participating in this trial receive the same anticancer treatment, the same number and type of visits and the same treatment for radiation dermatitis, oral mucositis and other toxicities as they would have received if not participating in the trial.

### Adverse event reporting and harms {22}

#### Assessment and documentation of adverse events

The severity of adverse events should be assessed using the Common Terminology Criteria for Adverse Events (CTCAE), version 5.0 [11]. Otherwise the following, the five-point scale below will be used to describe an adverse event: 1 = mild, 2 = moderate, 3 = severe, 4 = life-threatening, 5 = fatal

The following scale is used to describe the likelihood that the event was caused by the trial treatment:

1 = certain / definite, 2 = probable, 3 = possible, 4 = unlikely, 5 = not related, 9 = not assessable

Serious adverse events and unexpected adverse events must be reported within 24 hours after their detection/onset by fax to the coordinating investigator.

### Frequency and plans for auditing trial conduct {23}

The ZKS Lübeck will conduct on-site monitoring at the German sites according to principles of Good Clinical Practice (GCP) and written SOPs. For initiation, trial sites will be visited on-site. During the trial, sites will be visited at regular intervals depending on recruiting rate and data quality. Informed consent and defined key data will be checked of all patients. The medical file of each patient will be screened for adverse and serious adverse events. Patients’ questionnaires will be checked for their existence. Sites not based in Germany will be monitored according to their corresponding national regulations. No regular audits are planned but may be conducted if necessary. As this trial is not linked to the German pharmaceutical or medicinal product act, inspections of higher federal authorities are not planned.

### Plans for communicating important protocol amendments to relevant parties (e.g. trial participants, ethical committees) {25}

Amendments to the study protocol will be sent to and required approval from the local ethics committee. Only the coordinating principal investigator may carry out changes. If modifications appear required, co-investigators should contact the coordinating principal investigator. In the case of approved modifications of study protocol, all investigators will be informed.

### Dissemination of results and publication policy {31a, 32}

The coordinating principal investigator will strive for dissemination of trial results. Coordinating principal investigator and biostatistician will create a report according to the CONSORT statement, regardless of regular or early trial termination.

Results will be published in peer-reviewed journals and presented at scientific meetings. Reports and publications related to the trial must be coordinated with the biostatistician. Participating centers may use recorded data for additional analyses under their own name, but only after the main results have been published. Any additional or sub-publication requires approval by the coordinating principal investigator. For publications of any kind the study acronym DAI-BREAC must be used.

## Discussion

Adjuvant radiotherapy is an integral part of breast-conserving treatment of breast cancer patients and is also required after mastectomy in case of risk factors regarding loco-regional recurrences [1, 2]. During recent years, hypo-fractionated irradiation with a total dose of 40 Gy administered in 15 fractions of 2.667 Gy on 5 days per week is increasingly used [8, 9]. Ideally, radiotherapy is performed with precision techniques, namely intensity-modulated radiation therapy (IMRT) or volumetric modulated arc therapy (VMAT) [11]. Due to these techniques, the risk of radiation-related toxicities can be reduced. Another option that can help decrease the risks of cardiac or pulmonary morbidity deep-inspiration breath hold technique that is particularly used to treat left-sided breast cancer [14, 15]. Despite the improvement in radiation techniques, treatment-related adverse events still remain a risk in patients irradiated for breast cancer.

A common acute toxicity of radiotherapy for breast cancer is radiation dermatitis [3, 4, 12]. If dermatitis exceeds grade 1, it can be quite painful and have a negative impact on the patients’ quality of life [3]. If it becomes more severe, it may even require an interruption of the radiation treatment. In order to avoid grade ≥2 dermatitis, it is important that the patients perform specific skin care for the radiation fields several times per day, which should be started at the first day of radiotherapy. This may require considerable diligence and self-discipline from the patients. It may be questioned whether patients irradiated for breast cancer could benefit from a mobile application reminding them several times a day to perform their skin care. These reminders may improve the patients’ awareness and compliance. Improved adherence to the recommended skin care protocol may result in less radiation dermatitis.

In a previous retrospective study of our group that included 327 breast cancer patients irradiated in 2022 or 2023, 31.2% of the patients developed grade ≥2 radiation dermatitis [12]. Moreover, several factors showed highly or almost highly significant associations with the occurrence of grade ≥2 dermatitis on multivariate analysis, including history of chronic inflammatory disease (*p*=0.001), history of significant cardiovascular disease (*p*<0.001), smoking history of more than 10 pack years (*p*<0.001), normo-fractionated radiotherapy (*p*<0.001), and administration of a radiation boost (*p*<0.001). These risk factors were considered when designing the current DAI-BREAC trial. This randomized trial compares the rate of grade ≥2 radiation dermatitis with standard skin care plus a reminder app to the dermatitis rate with standard skin care alone in a cohort of 268 patients. If it is demonstrated that the reminder app contributes to a clinically relevant reduction of radiation dermatitis in breast cancer patients, such an app will likely become a useful tool for this group in the near future. When the final results of the DAI-BREAC trial will be available, a limitation of this trial should be considered, namely the fact that standard skin care practices may vary between the contributing centers. This article considered the items recommended to address in the SPIRIT 2013 checklist [16–18].

### Trial status

Protocol version 1.2 from 21-JUN-2024, recruitment started in December 2024 and will be completed within 24 months.

## Data Availability

The trial was registered at clinicaltrials.gov (identifier: NCT06483477), where details of the study protocol reported in this article are available. Study results will be made available at clinicaltrials.gov as well and published in a peer-reviewed scientific journal.

## Abbreviations

CRF: Case Report Form
CTCAE: Common Terminology Criteria for Adverse Events
ECOG: Eastern Cooperative Oncology Group
ECOG-PS: Eastern Cooperative Oncology Group Performance Status
eCRF: Electronic Case Report Form
DIBH: Deep-Inspiration Breath Hold
GCP: Good Clinical Practice
GDPR: General Data Protection Regulation
HeAT: Health Advancing Technologies for elderly
HER2: Human Epidermal growth factor Receptor 2
IMRT: Intensity-Modulated Radiation Therapy
OHG: Offene Handelsgesellschaft - General Commercial Partnership
SAP: Statistical Analysis Plan
VMAT: Volumetric Modulated Arc Therapy
ZKS: Zentrum für Klinische Studien - Centre for Clinical Trials
